# The role of social motivation in sharing and fairness: insights from Williams syndrome

**DOI:** 10.1186/s11689-024-09568-3

**Published:** 2024-08-31

**Authors:** Francesca Foti, Floriana Costanzo, Carlo Fabrizio, Andrea Termine, Deny Menghini, Tiziana Iaquinta, Stefano Vicari, Laura Petrosini, Peter R. Blake

**Affiliations:** 1 Department of Educational Sciences, University of Catania, Catania, Italy; 2 Child and Adolescent Psychiatry Unit, Bambino Gesù Children’s Hospital, IRCCS, Rome, Italy; 3 Santa Lucia Foundation IRCCS, Rome, Italy; 4 Department of Medical and Surgical Sciences, “Magna Graecia” University of Catanzaro, Catanzaro, Italy; 5 Department of Life Sciences and Public Health, Catholic University, Rome, Italy; 6 Department of Psychological & Brain Sciences, Boston University, Boston, MA USA

**Keywords:** Williams syndrome, Developmental disorders, Dictator game, Inequity game, Inequity aversion, Social cognition, Behavioral phenotype, Social phenotype, Fairness, Resource distribution, Children

## Abstract

**Background:**

Sharing and fairness are important prosocial behaviors that help us navigate the social world. However, little is known about how and whether individuals with Williams Syndrome (WS) engage in these behaviors. The unique phenotype of individuals with WS, consisting of high social motivation and limited social cognition, can also offer insight into the role of social motivation in sharing and fairness when compared to typically developing (TD) individuals. The current study used established experimental paradigms to examine sharing and fairness in individuals with WS and TD individuals.

**Methods:**

We compared a sample of patients with WS to TD children (6-year-olds) matched by mental age (MA) on two experimental tasks: the Dictator Game (DG, Experiment 1, *N* = 17 WS, 20 TD) with adults modeling giving behaviors used to test sharing and the Inequity Game (IG, Experiment 2, *N* = 14 WS, 17 TD) used to test fairness.

**Results:**

Results showed that the WS group behaved similarly to the TD group for baseline giving in the DG and in the IG, rejecting disadvantageous offers but accepting advantageous ones. However, after viewing an adult model giving behavior, the WS group gave more than their baseline, with many individuals giving more than half, while the TD group gave less. Combined these results suggest that social motivation is sufficient for sharing and, in particular, generous sharing, as well as the self-focused form of fairness. Further, individuals with WS appear capable of both learning to be more generous and preventing disadvantageous outcomes, a more complex profile than previously known.

**Conclusions:**

In conclusion, the present study provides a snapshot into sharing and fairness-related behaviors in WS, contributing to our understanding of the intriguing social-behavioral phenotype associated with this developmental disorder.

**Supplementary Information:**

The online version contains supplementary material available at 10.1186/s11689-024-09568-3.

## Background

Sharing and fairness are important behaviors for navigating the social world and managing relationships with others. Typically developing (TD) children readily engage in simple forms of both sharing and fairness from an early age and much research has examined the development of these behaviors through the school years. However, less is known about the how these phenomena appear in atypical populations. For example, only two studies have tested sharing and fairness in children with autism spectrum disorder (ASD) [1, 2], but no studies have tested individuals with Williams syndrome (WS). The population with WS shows a profile of high social motivation and deficits in social cognition which allows examining the unique role of these constructs in sharing and fairness behaviors. Thus, investigating these behaviors can not only elucidate the behavioral phenotype for this neuro-developmental disorder, but also deepen our understanding of the psychological components required for sharing and fairness.

### Williams syndrome

Williams syndrome is a rare genetic disorder with an incidence rate of one in 7000 live births [3, 4]. The classic cognitive-behavioral phenotype of WS is characterized by mild to moderate intellectual disability [5, 6], severe visuospatial alterations [7–11], and a high drive toward social engagement even with strangers [12–14]. Individuals with WS typically demonstrate an overly friendly, affectionate, engaging, and socially disinhibited personality [15–17]. However, the social behavior of individuals with WS is often inappropriate and is accompanied by marked deficits, such as difficulties in social adjustment, social judgment, and practical social interactions [18, 19]. These deficits make it difficult for individuals with WS to form friendships and navigate peer relations. Overall, the social phenotype of WS has been described as high in social motivation coupled with deficits in social cognition [20, 21].

To clarify the distinction between social motivation and social cognition, we offer the following definitions. Social motivation refers to both the desire to engage in social interactions and the reward value of social interactions. In the latter case, social interactions may have higher inherent value compared to other rewards such as material goods or monetary compensation as typically used in economic games. Social motivation is a broad description that can include both prosocial (positive) and antisocial (negative) behaviors towards others.

Social cognition is also a broad description that refers to a range of capabilities including the ability to perceive, represent, process and reason about social situations. Social cognition allows individuals to understand and predict the actions of others and facilitates engagement with others. Although other definitions are possible, the distinction here explains a range of evidence described next.

The combination of high social motivation and lower social cognition compared to TD populations results in an uneven profile of abilities for individuals with WS. For example, multiple studies have found that individuals with WS are successful on simple perceptual tasks that test theory of mind abilities, including matching photos of faces expressing emotions [17], reading the mind in the eyes [22], imitating emotional expressions [23], as well as on story sequence tasks that test understanding of intention and pretense [24]. However, most studies testing samples with WS on false belief tasks (i.e., change of location, change in contents) find deficits, suggesting some difficulties in representations related to social cognition [17, 24].

For social learning, individuals with WS perform similarly to TD individuals on imitation tasks. For example, in a pattern-finding task, both WS and TD samples improved after observing an experimenter find the pattern [25, 26]. WS children are also similar to TD children on overimitation. In a container-opening task, both groups engaged in more causally irrelevant imitation and looked more at the demonstrator’s face compared to ASD children [27]. Given difficulties with understanding the causal-intentional nature of actions [28], the performance of individuals with WS on imitation tasks provides evidence in support of social-motivational accounts of overimitation as opposed to social-cognitive accounts [29].

Of particular relevance to the current study, individuals with WS show high to typical levels of prosociality which depends on social motivation. Parental report measures reveal distinctly high levels of empathy [30] and strengths for prosocial behaviors, such as showing concern for others and congratulating others on accomplishments [18]. Experimental studies have also found that children with WS engaged in more empathic comforting behaviors when an experimenter feigned a hurt knee compared to children with Down syndrome (DS) and TD children [31]. In the same study, children in all groups engaged in spontaneous helping when the experimenter accidentally spilled beads on the floor, but children with WS helped less compared to TD children when the experimenter asked for help folding a toy tunnel, a task requiring more cognitive and visual-motor abilities.

Combined these studies show that the social phenotype for WS consists of high social motivation and lower social cognitive abilities compared to TD children. This general pattern applies across social behaviors from theory of mind to social learning and simple forms of prosociality. Importantly, comparisons of populations with WS to TD children matched on mental age has revealed that many basic skills of social interaction do not require sophisticated representational and cognitive abilities but rather rely more on social motivation. We next turn to sharing and fairness to consider the role of social motivation for both.

### Social motivation for sharing

Research on the development of sharing has found that this form of prosociality emerges very early. Toddlers engage in acts of sharing with adults [32, 33] and by 3–4 years of age children share some items with peers when they receive a windfall gain of resources [34–39]. Notably, although preschoolers give more on average with age, they still share less than half with peers in most circumstances [40].

Multiple cognitive processes appear to underlie the development of sharing. For example, several studies show that sharing requires behavioral control [41, 42] and is associated with better performance on executive function tasks [43, 44]. Some consideration of the recipient is also needed [45, 46], but theory of mind (false belief in particular) does not seem to be a pre-requisite [44, 47]. In addition to these cognitive functions, some have claimed that sharing is intrinsically motivated for children as young as 3 years of age [48]. According to this account, sharing is similar to other prosocial behaviors like helping and comforting that occur spontaneously in toddlers [49, 50].

One way to assess the role of social motivation in sharing behavior is to test participants with WS. Given their high levels of social motivation, a group with WS might give more than TD controls in sharing tasks. However, it is also possible that high social motivation does not increase prosociality beyond that of TD controls, as has been found for helping tasks [31]. Given this possibility, an alternative test is to have an adult model giving behavior that is rare among TD children – giving more than half – and then test whether the participants will show similarly generous behavior. A version of this task conducted with children in both the US and rural India found that children in the US who saw the generous model before they shared with a peer did not give more than half but children in India did [51]. This suggests that children in the US, and possibly other Western societies, may have limited social motivation to learn from the adult when actions are costly to them. Because populations with WS may have greater social motivation to learn from the adult in this context, the modelled giving task offers a strong test of the role of social motivation in costly giving.

### Social motivation for fairness

Fairness, in its most basic form, involves an equitable distribution of resources between two or more individuals [52]. For the case of windfall gains, when none of the potential recipients of resources is more deserving or has greater need, equality is the most straightforward solution. Multiple studies have shown that infants expect equal outcomes in social contexts but not more generally [53–55]. By 3–4 years of age, children are upset when receiving less than a peer, but tend to maintain an advantage when distributing resources themselves [39, 56]. This general pattern continues into middle childhood at which point children will choose equality over an advantage [57].

As with sharing, multiple cognitive processes are involved in decisions about fairness. First, one must engage in social comparison and quantitative comparison. This may require perceptual or surface comparisons, but also requires attention to what the other person has or will receive and not just one’s own gains. Second, some motivation to decrease inequality must be present. This social motivation for fairness is distinct from the more general prosocial motivation that is required for sharing [52]. For example, in the sharing tasks with generous models described above, a children can be very generous, giving more than half to a peer, but doing so will increase inequality. Given the difficulty of teasing these two motivations apart, a test for fairness should allow one to decrease inequality in a way that is not prosocial. Third, one must inhibit self-interest in order to give up resources to minimize inequality, particularly when one has an advantage.

Theory of mind abilities are also associated with fairness preferences in TD children but this depends on the tasks used [58]. For example, performance on false belief tasks is associated with children’s decisions in the Ultimatum Game, a fairness and bargaining task that requires strategic thinking [59]. In the Ultimatum Game, one player proposes a division of resources between themselves and a peer, and then the peer then decides to accept the offer or to reject it, in which case neither player gets anything. Rejections of unequal divisions are understood as protests against unfairness. By about 5 years of age, TD children reject unequal offers in this task [59, 60]. However, the bargaining aspect of the task requires sophisticated social cognitive skills in order to understand what alternatives are possible and what the proposer intends. In fact, studies comparing TD and ASD children in Ultimatum Games have found that ASD children are much more likely to accept low offers including offers where the receiver gets nothing [1, 2].

An alternative to the Ultimatum Game is the Inequity Game (IG) which assesses fairness by focusing on responses to inequality itself, a more simple cognitive task. In the IG, the experimenter presents an allocation of resources to two children and one of the children decides whether to accept the distribution or reject it in which case neither child gets anything [61]. Multiple studies using IG have demonstrated that children as young as 4 years of age will reject a disadvantageous offer and accept an advantageous offer. This result has been found in eight countries to date, including non-Western populations, such as villages in rural India, rural Uganda and rural Peru, a city in Senegal and two locations in China [62–64]. By about 9 years of age, children in the US, Canada, Uganda and China also reject an advantage (four treats when the peer will receive only one), thus making a large sacrifice to prevent a peer from receiving less. Although the IG is typically conducted with two children in-person, related tasks have obtained similar results using recipients that are hypothetical [65], presented in a photo [66] and puppets [67]. Combined these studies have established the IG as a valid and standard test for fairness.

Rejecting disadvantageous inequality requires a social motivation that depends on social comparison but is self-focused. Researchers have argued that an aversion to advantageous inequality requires more sophisticated social cognition including an integration of social norms and behavioral control [57], feelings of guilt and empathy [68], and concerns for one’s reputation [69]. Although a social motivation to create fairness for others is also central to advantageous inequity aversion, it remains unknown whether this is sufficient to demonstrate this more robust form of fairness. To date, no studies have assessed the disadvantageous and advantageous forms of fairness with populations with cognitive deficits. Testing WS populations using the Inequity Game can thus offer insights into the role of social motivation for both forms of fairness.

### The present study

In the current study, we tested the role of social motivation in both sharing (Experiment 1) and fairness (Experiment 2). We tested a patient population of individuals with WS and compared them to a mental age- and sex-matched sample of TD children. To test sharing, we used both a standard giving task, the Dictator Game (DG) and a version in which adults modeled a generous or selfish level of giving in the DG. For the standard DG, we predicted that the WS group would give more than the TD group. For the modelled DG in the generous condition, we predicted that the WS group would give more compared to the baseline for their group, and more compared to the TD group in the generous condition. We also predicted that the TD group would not give more than their baseline in the generous condition, mirroring results found in the US in a similar experiment. For the modelled DG in the selfish condition, we predicted that both the WS and TD groups would give less than their baseline DGs, but we did not predict a difference between groups. The pattern for the TD group would thus mirror what was found with a US sample [51]. We also planned secondary analyses to determine whether there were differences between and within groups for giving zero, giving half and giving more than half. Our only prediction for these analyses was that the WS group would be more likely to give more than half in generous condition compared to the baseline condition and compared to the TD group in the generous condition.

For fairness, we used the Inequity Game, testing both the disadvantageous and the advantageous conditions for both WS and TD groups. We predicted that the TD group would show the pattern that has been found in other Western societies: rejecting disadvantageous offers and accepting advantageous offers (typical for 6-year-olds). For the WS group, we predicted that social motivation, and prosocial motivation in particular, would lead participants to accept disadvantageous offers. The reason is that accepting is generous in this case: the recipient gets four treats and the participant gets one. For advantageous offers for the WS group, our predictions were less clear. Social motivation is needed to reject the offer and deny oneself an advantage. However, greater social cognition is required to interpret rejecting as nice in this case. One must recognize that the recipient may be disappointed if they receive less than the participant and therefore that rejecting the offer and creating equality (nothing for both) is the fair solution. Given the combination of high social motivation and deficits in social cognition, we did not have a strong prediction for the WS group in the advantageous condition. However, if the WS group did reject advantageous offers, this would provide evidence that social motivations may be sufficient for this form of fairness.

## Methods

### Participants for both experiments

All participants with WS were clinically diagnosed and the diagnosis was confirmed by fluorescence in situ hybridization (FISH) genetic investigation, which showed the characteristic deletion on chromosome band 7q11.23. Participants with WS were part of a larger pool of individuals with learning disabilities attending the local hospital where the study was conducted for clinical follow-up. The TD participants were recruited from local schools in the same area. To compute Intelligence Quotient (IQ) and the corresponding Mental Age (MA) of all participants we used the Leiter International Performance Scale–Revised [70]. All participants were tested on both the Dictator Game (DG) and the Inequity Game (IG) on different days. The order of the tasks was counterbalanced, but preliminary assessments of the data showed that there were no order effects and this variable is left out of the analyses that follow.

This study was approved by the Ethics Committee of Children’s Hospital “Bambino Gesù,” Rome, Italy and conducted according to the Helsinki declaration. The parents of all individuals who participated in the study provided written informed consent. The study was performed in Italy.

### Experiment 1: Dictator game

All participants received the standard DG followed by both the Generous and Selfish model conditions with order counterbalanced between participants.

## Method

### Participants

Seventeen individuals with WS and 20 TD children matched for MA and gender participated to DG (Table 1). The WS and TD groups differed in chronological age (CA) [Welch’s T-test: *T* (16.15) = -6.05; *p* < 0.001] and IQ [Welch’s T-test: *T* (32.24) = 16.31; *p* < 0.001] but not in MA [Welch’s T-test: *T* (29.07) = 0.63; *p* = 0.53].

**Table 1:** Age and intelligence characteristics by task and group

Sample characteristics					
Task	Group	Gender	Chronological Age (in years) mean ± SD median, q_1,_q_3_ (range)	IQ mean ± SD median, q_1,_q_3_ (range)	Mental Age (in years) mean ± SD median, q_1,_q3 (range)
**Dictator Game**	WS	9 M 8 F	22.0 ± 10.0 20, *q*_*1*_ = 14.3, *q*_*3*_ = 29.11 (range: 8–41.09)	47.9 ± 11.0 44, *q*_*1*_ = 38, *q*_*3*_ = 56 (range: 36–68)	5.93 ± 1.27 5.6, *q*_*1*_ = 5.10, *q*_*3*_ = 7 (range: 4.01–8.07)
	TD	11 M 9 F	6.05 ± 0.74 6.11, *q*_*1*_ = 5.70, *q*_*3*_ = 6.65 (range: 4.07–7.04)	104.0 ± 9.67 102.50, *q*_*1*_ = 97, *q*_*3*_ = 108 (range: 89–121)	6.17 ± 0.94 6.10, *q*_*1*_ = 5.63, *q*_*3*_ = 6.73 (range: 4.09–7.09)
**Inequity Game**	WS	7 M 7 F	23.1 ± 10.09 21.05, *q*_*1*_ = 16.58, *q*_*3*_ = 29.11 (range: 8–41.09)	48.7 ± 5.05 47, *q*_*1*_ = 37, *q*_*3*_ = 59 (range: 36–68)	6.15 ± 0.77 5.85, *q*_*1*_ = 5.11, *q*_*3*_ = 7.08 (range: 4.05–8.07)
	TD	9 M 8 F	5.98 ± 0.08 6, *q*_*1*_ = 5.7, *q*_*3*_ = 6.2 (range: 4.07–7.04)	103 ± 3.78 100, *q*_*1*_ = 97, *q*_*3*_ = 107 (range: 89–121)	6.03 ± 0.14 6, *q*_*1*_ = 5.4, *q*_*3*_ = 6.7 (range: 4.09–7.09)

### Procedure

**Fig. 1:**
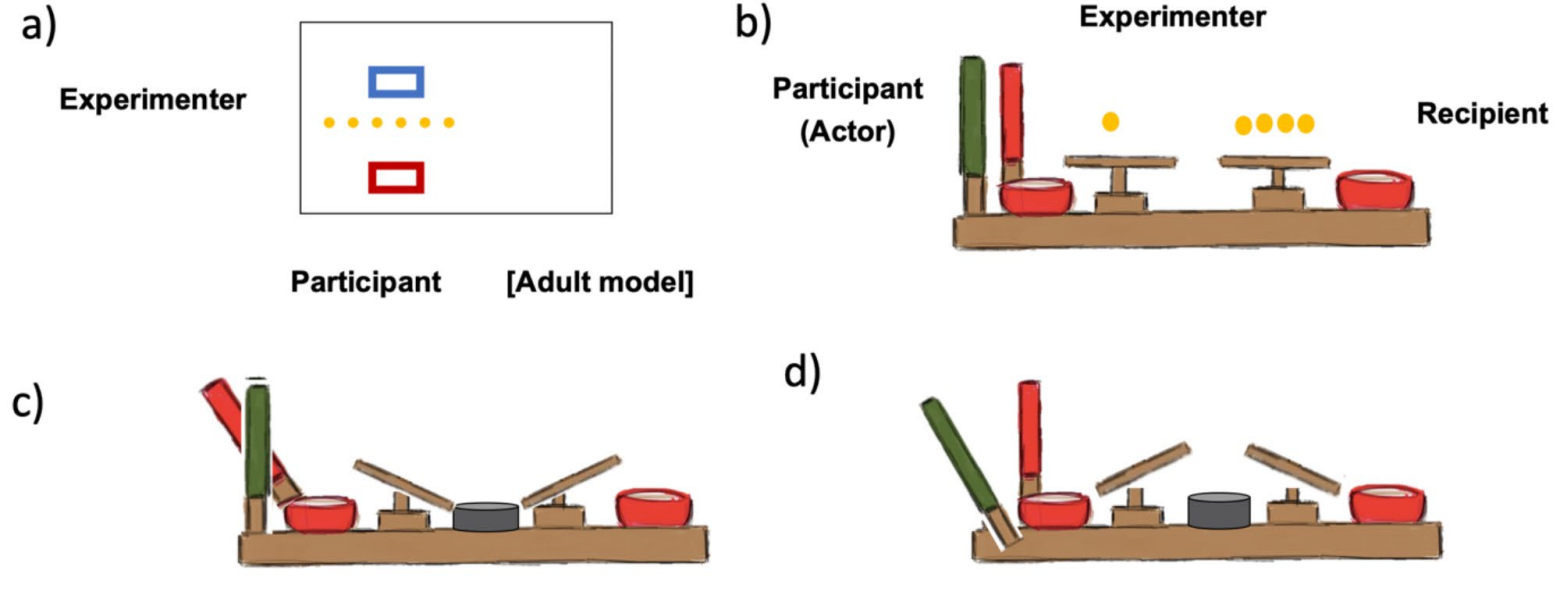
Set-up and apparatuses for Experiments 1 and 2. (a) Dictator Game. The experimenter was seated on one side of the table and arranged one row of 6 candies in front of the participant. The participant had a red box in front of him/her where s/he put the candies to bring home, and a blue box where s/he put the candies to donate to the recipient. The adult model sat next to the participant so that the participant saw the allocations made by the model from the same perspective. (b) Inequity Game set up. The participant sat on the side with the handles and the recipient was across from them; the experimenter was at the side, placing treats on the trays. The participant could pull the red handle (c) to reject an allocation so that all treats went into the middle bowl and no one received anything, or they could pull the green handle (d) to accept the allocation so that the treats went into the outer bowls for the participant and the recipient

The Dictator Game involved an allocator (WS or TD participant) and a non-present receiver gender-matched with the participant. Children were told that the experimenter would see the recipient later. Participants were tested individually at a small rectangular table (Fig. 1a). Two boxes, one red and one blue, were on the table in front of the participant, with the red box closest to the participant.

Participants received three trials and in each trial 6 candies were used. The first trial was the baseline trial. The experimenter placed one row of 6 candies in front of participant and said: “*Right now all of these candies belong to you. You can do whatever you want. You can keep all of them for yourself or you can give some to another boy/girl who I will see later. I’ll make sure that the boy/girl likes the candies. Any candies that you want to keep for yourself*,* you can put into red box and any that you want to give to the other boy/girl you can put into blue box. And in a minute*,* I’m going to turn around*,* so no one can see what you decide to do. It will be up to you.*” After the instructions, the participant performed the task.

For the next two trials, the participant watched the adult model sitting next to him/her who performed the task and allocated specific amounts of rewards between herself and the receiver. Specifically, the participants watched two donation models on separate trials: a selfish donation, in which the adult model gave 1 out of 6 candies to the recipient and kept 5 candies for herself, and a generous donation, in which the adult model gave 5 out of 6 candies to the recipient and kept 1 candy for herself. The adult model turned away from the game after each donation demonstration. After watching each donation model, the participant did the task. The order of the Selfish and Generous model trials was counterbalanced among participants. The experimenter specifically told the children that there was a different recipient for each trial.

After each trial, the contents of the red and blue boxes were emptied into paper bags, one for the participant and one for the receiver.

### Statistical analyses

The statistical analyses were performed in R statistical software (version R 4.0.3, 2020, The R Foundation for Statistical Computing). Preliminary tests showed that the dependent variable (number of candies given to the recipient) was not normally distributed: Shapiro-Wilk test, *W* = 0.927, *p* = 0.018. Therefore, the total number of rewards given to the recipient between groups was analyzed using a heteroscedastic one-way ANOVA for medians [71], using the WRS2 package [72]. The main effect and interactions were plotted using the ‘sjPlot’ package [73]. Model exposure effects in the two groups were tested using Friedman tests [74] and subsequent Conover post-hoc tests [75] using the ‘PMCMR’ package [76].

We used a linear mixed effects models (‘nlme’ package) in a preliminary analysis to determine whether model order improved data fit over an intercept-only model. Model order did not improve the fit (χ^2^ (3,4) = 0.01, *p* = 0.936) and was not included in subsequent analyses.

## Results

A heteroscedastic one-way ANOVA for medians on the number of rewards given to the recipient revealed a significant difference between groups (*F* = 5.18; Critical value = 3.57; *p* = 0.022). The WS group had a higher median level of giving across all conditions (median = 3) compared to the TD group (median = 2). We next used Wilcoxon rank sum tests to determine whether there were differences between groups for each condition. The WS and TD groups did not differ in the Baseline condition (*W* = 191, *p* = 0.503) or in the Generous condition (*W* = 224, *p* = 0.093) but the WS group gave more in the Selfish condition (*W* = 234, *p* = 0.046). Overall, the WS group gave more in the modeling conditions compared to the TD group.

**Fig. 2:**
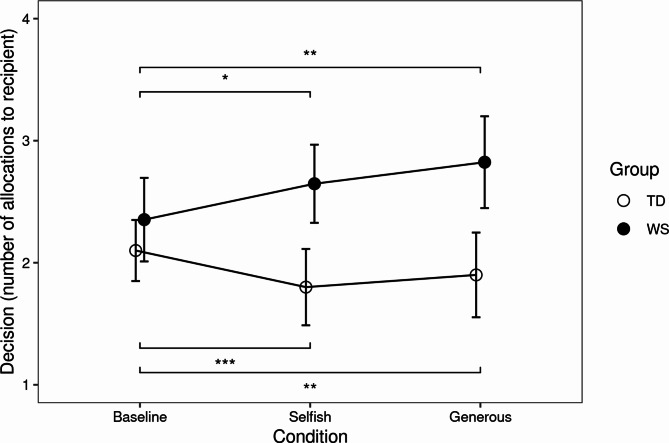
Dictator Game. The average number of items (out of 6) that were donated to the recipient, by group (TD and WS) and by condition (Baseline, Selfish and Generous). Error bars are standard errors. The TD and WS groups gave similar amounts in the Baseline condition and deviated from Baseline after exposure to the Selfish and Generous models (* *p* < 0.05, ** *p* < 0.01, *** *p* < 0.001)

We predicted that within each group, giving in the modeling conditions would differ from the Baseline condition (Fig. 2). For both groups, Friedman tests showed an overall difference in amount given across Conditions: WS: Friedman χ^2^(2) = 77.64; *p* < 0.001; TD: Friedman χ^2^(2) = 90.79; *p* < 0.001). Follow-up comparisons were performed using the Conover test with Holm adjustment. For the WS group, compared to the Baseline trial, participants gave significantly more in both the Selfish trial (*p* < 0.05) and the Generous trial (*p* = 0.01), but there was no difference between the Selfish and Generous trials. For the TD group, compared to the Baseline trial, participants gave significantly less in both the Selfish trial (*p* < 0.001) and the Generous trial (*p* < 0.01), but there was no difference between the Selfish and Generous trials.

As a secondary analysis, we examined changes in categories of donations levels based on prior research with children using the Dictator Game [36]. For each group, we compared donations in the Baseline trial to donations in the Selfish and Generous trials for each donation level. We used generalized linear mixed effects models (‘glmer’ function) to model whether the main effects of Group and Condition and their interaction predicted each of three dichotomous outcomes: Give Zero, Give Half and Give more than Half. The only significant effect emerged for the Give more than Half model, with the WS group more likely to give over half compared to the TD group (*β* = 1.57, *SE* = 0.73, *p* = 0.034). There was also a trend for participants to give more than half in the Generous condition compared to the Baseline condition (main effect: *β* = 1.75, *SE* = 0.98, *p* = 0.073). This trend appeared in both groups (TD: Baseline 0%, Generous 10%; WS: Baseline 12%, Generous 29%).

We also conducted a correlation analysis for MA and CA and number of resources shared by Group and Condition (Table 2). There were no significant correlations with CA and only one significant correlation with MA. For the TD group, Baseline trial, resources shared increased with MA (*R* = 0.45, *p* = 0.05).

**Table 2:** Dictator game, correlations with Chronological and Mental age. A Correlations between chronological age and the number of items donated to the recipient for each group. B Correlations between mental age and the number of items donated to the recipient for each group

Group	Baseline	Selfish	Generous
**A) Chronological Age**			
WS	*R* = 0.01; *p* = 0.97	*R* = 0.09; *p* = 0.74	*R* = 0.24; *p* = 0.36
TD	*R* = 0.29; *p* = 0.21	*R* = 0.09; *p* = 0.72	*R* = 0.18; *p* = 0.44
**B) Mental Age**			
WS	*R* = 0.17; *p* = 0.5	*R* = 0.11; *p* = 0.7	*R* = 0.42; *p* = 0.09
TD	***R*** **= 0.45; *p*** **= 0.05**	*R* = -0.11; *p* = 0.65	*R* = -0.08; *p* = 0.72

## Discussion

The results of the Dictator Game experiment revealed both similarities and differences in how participants with WS and the TD sample behaved in the three conditions (Baseline, Selfish, and Generous). There were three key results. First, participants in the WS group gave more overall compared to the TD group, but contrary to our predictions the WS group did not give more than the TD group in the Baseline condition. In the modelling conditions, the WS group gave more in the Selfish condition than the TD group, but only showed a non-significant trend for giving more compared to the TD group in the Generous condition. We predicted that within each group, giving in the modelling conditions would deviate from that group’s baseline giving. For the Generous model condition, the WS group gave more compared to baseline, which we had predicted, but the TD group gave less than their baseline. For the Selfish model condition, the TD group gave less compared to their baseline, which we had predicted. However, the WS group actually gave more than their baseline in the Selfish model condition.

Lastly, we predicted that the WS group would be more likely to give more than half in Generous condition compared to baseline and TD. Although 29% of the WS sample gave more than half in the Generous condition compared to 10% of the TD group, this was not a significant difference. However, the WS group was more likely to give more than half compared to the TD group across all conditions combined.

In summary, the WS participants did not share differently from the TD group in the Baseline condition. However, they did share more compared to the TD group after observing an adult model give any amount to a recipient, and they were more willing to give more than half. Combined these results show that individuals with WS are willing to be more generous after watching others give and suggests that social motivations may be the main driver of generosity.

### Experiment 2 - Inequity game

Participants played the Inequity Game in the actor role in both the Disadvantageous and the Advantageous conditions in two different testing sessions.

## Methods

### Participants

Fourteen individuals with WS and 17 TD children participated in the IG (3 individuals with WS did not participate in or complete the task). Participants were matched for MA and gender (Table 1). The WS and TD groups differed in CA [Welch’s T-test: *T* (16.15) = -6.54; *p* = 0.006] and IQ [Welch’s T-test: *T* (32.26) = 16.31; *p* < 0.001] but not in MA [Welch’s T-test: *T* (29.07) = 0.63; *p* = 0.53]. Gender balance of the two groups was similar (Fisher’s exact test, *p* = 1).

### Apparatus

We used the IG apparatus that has been used in multiple studies [61, 62]. The apparatus had two trays, one closer to the participant (actor), who could accept or reject the allocation of rewards, and one closer to the “recipient”, who played a passive role (Fig. 1b). The trays and the handles were attached to a plywood board and connected with fishing wire. When the participant pulled the green handle (accept), the trays tilted outward so that the rewards placed on them fell into the participant’s and recipient’s bowls (Fig. 1d). When the participant pulled the red handle (reject), the trays tilted towards the center so that the rewards from both trays fell into the middle bowl (Fig. 1c). To indicate when the trial would start, the experimenter placed a wooden stick, approximately 24 centimeters in length and half a centimeter in diameter, on the trays in between trials and removed it to signal that the participant could now decide which handle to pull.

### Experimental procedure

Each participant (the actor) performed the task face-to face with the recipient (Fig. 1b). The recipient was a puppet managed by the experimenter. Puppets have been used as proxies for peers in dozens of developmental psychology studies [77–81] including experiments on resource decisions [81–84]. Puppets have also successfully been used in studies with individuals with autistism [85] and Williams syndrome [86, 87]. In general, participants are more willing to criticize or protest the actions of puppets compared to actual peers or adults. A further consideration for the IG task is that participants are usually matched with same-age, same-gender peers. However, the wide age range of the WS group made this impractical. The puppet recipient allowed us to standardize the experiment across both TD and WS groups.

The participant controlled the pair of handles, which were used to enact decisions, and the recipient played a passive role. A second experimenter placed candy rewards on both sides of the apparatus, always placing the rewards on the recipient’s side first in order to ensure that the participant paid attention to the recipient’s payoff before attending to their own. Before starting the game, the experimenter demonstrated how the handles worked in two trials with one candy on each tray. The experimenter showed the participant how they could accept or reject the allocation of the rewards by pulling the green or the red handle, respectively. Participants were told that the rewards that fell into the outer bowls could be taken home at the end of the game by them and the recipient, respectively. They were told that neither s/he nor the recipient could take the rewards fell into the middle bowl.

After the game explanation, the participants were given three practice trials to ensure that they understood the game and the effects of pulling handles. Participants were given no feedback on the decisions made in these trials. The practice trials were as follows: 1–1 (equal: 1 reward for the actor, 1 reward for the recipient); 0–1 (disadvantageous inequity: 0 for actor, 1 for recipient) and 1 − 0 (advantageous inequity: 1 for actor, 0 for recipient). If the participant pulled the same handle on all three trials, an additional trial of 1–1 was given and the participant was told to pull the other handle “just to see what will happen.”

Each participant did two conditions of the Inequity Game, disadvantageous and advantageous, during two separate sessions. The order of the conditions was counterbalanced among participants. In the disadvantageous condition, there were 6 equal trials (1 reward each to the actor and the recipient) and 6 unequal trials (1 reward for the actor, 4 rewards for the recipient). The order of the equal and unequal trials was randomized. The advantageous condition was similar except that in the 6 unequal trials the actor received 4 rewards and the recipient received 1 reward.

For the 12 test trials in each condition, the experimenter held the wooden stick on the trays to set them level, placed the rewards, and then lifted the stick, at which point the participant could pull one of the handles. If the participant did not pull a handle within 5 s, the experimenter placed the stick on the trays again and asked, “*Which handle do you want to pull?*” and then lifted the stick. After each decision, the experimenter stated the outcome (e.g., “*You [participant] get one and [recipient] gets four*,” *“No one gets anything that time”*). After all the trials were completed, the experimenter said that the task was over, and that the participant could put his/her candy into a paper bag.

The standard experimental procedure used with neurotypical children has several features that decrease the cognitive demands for participants. First, all participants see a demonstration of how the handles work and where the candies fall when the allocation is accepted or rejected. They then receive practice trials to ensure that they experience the consequences of pulling each of the handles. Second, for each trial the experimenter places the rewards on the recipient’s side first and then the participant’s side and states the amount each time. This facilitates both social comparison and quantitative comparison. Third, the unequal reward amounts are 1 vs.4, a relatively large difference that is visually obvious and limits the need for counting. Fourth, at the start of each trial, the experimenter places the candies while holding the trays flat with a stick, then lifts the stick and asks the participant to make a decision. This helps to focus attention for each trial and prompts decision making. These procedures gave us confidence that the WS sample would be able to understand and complete the task despite any cognitive deficits.

Rejections were used as the dependent variable because children must deny themselves and the recipient rewards when rejecting. Rejections thus go against self-interest and are also not prosocial. Although any rejections are of interest, we compare rejections in the unequal trials to those in the equal trials to determine whether a group shows each form of fairness: disadvantageous and advantageous. This approach allows us to control for random behavior such as alternating pulling each handle. If a participant did this, then they would reject half of the equal and half of the unequal trials, treating both types of trials the same. However, if a participant rejects significantly more unequal than equal trials, this is not a random pattern.

### Statistical analyses

All statistical analyses were performed in R statistical software (version R 4.0.2, 2020 The R Foundation for Statistical Computing). Data were analyzed using generalized linear mixed models (GLMMs) with a binary response term (0 = accept, 1 = reject). All models were run by using the package “lme4” [88] with subject’s id code as random effect. Following standard analyses using this task [62, 89], we compared a null model with only intercept terms (including the random effect) to a full model using likelihood ratio tests (LRT). The initial full model included our primary variables of interest: Distribution (Equal, Unequal), Condition (Disadvantageous, Advantageous), Group (WS, TD) and all interaction terms. The full model provided a better fit to the data (*LRT* χ^2^ (7) = 21.763, *p* = 0.003). We examined the interaction between Distribution and Condition to determine whether to split the sample by condition, following the standard analyses for this task. The interaction was significant (*β* = 1.223, *p* = 0.005) and all subsequent analyses were performed on subsets of the data for the Disadvantageous and Advantageous conditions. We added design variables (Order of conditions, Trial number, Session number) and covariates (MA, IQ) in blocks to each subset analysis to determine whether they improved the fit to the data. We used this approach to avoid overfitting given the relatively small number of participants.

## Results

**Fig. 3:**
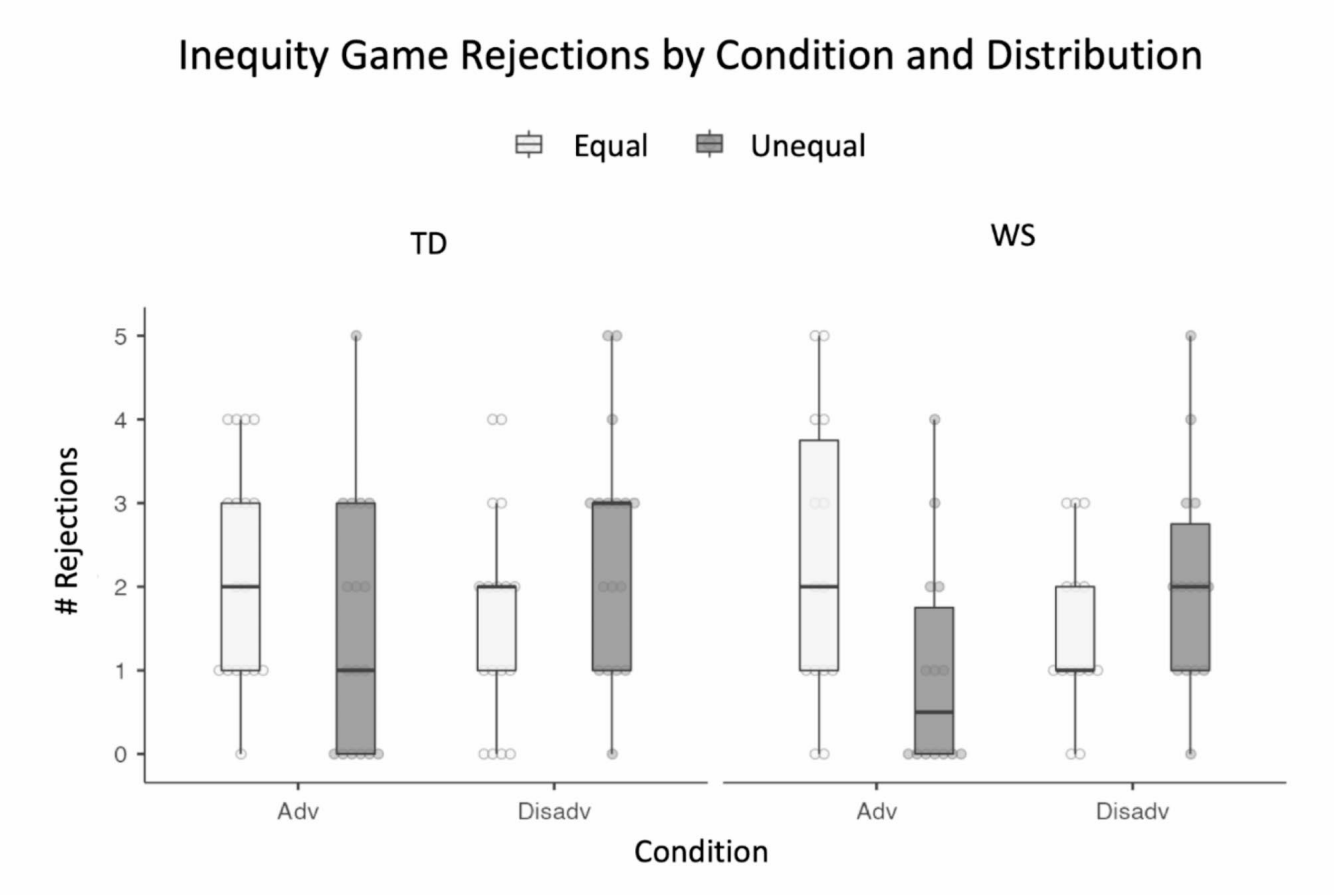
Inequity game rejections by condition and distribution. Boxplot of rejections of equal and unequal trials in the advantageous and disadvantageous conditions for the TD and WS groups

For each condition, we compared an intercept only model to a model with the primary variables of interest: Distribution and Group. We tested both interaction models and main effects models. For the Disadvantageous condition, the interaction model did not improve the model fit compared to the intercept only model (*LRT* χ^2^ (3) = 6.95, *p* = 0.073), but the main effects model did (*LRT* χ^2^ (2) = 6.84, *p* = 0.033). We next added the two blocks of variables to the main effects model but neither block further improved the model fit. The final model revealed higher rejections of the unequal trials compared to the equal trials (*β* = 0.547, *p* = 0.015), but no significant difference between the WS and TD groups (Fig. 3). Thus, contrary to our predictions, participants in both groups made similar decisions, rejecting disadvantageous trials more than equal distribution trials.

For the Advantageous condition, the interaction model significantly improved the fit compared to the intercept only model (*LRT* χ^2^ (3) = 15.00, *p* = 0.002), but the interaction term was not significant. We next tested the main effects model which also improved fit over the intercept only model (*LRT* χ^2^ (2) = 13.35, *p* = 0.001). The interaction model did not improve the fit to the data compared to the main effects model so the more simple model was used as the base. We next added the two blocks of variables to the main effects model but neither block further improved the model fit. The final model revealed lower rejections of the unequal trials compared to the equal trials (*β* = -0.874, *p* < 0.001), but no significant difference between the WS and TD groups (Fig. 3).

**Fig. 4:**
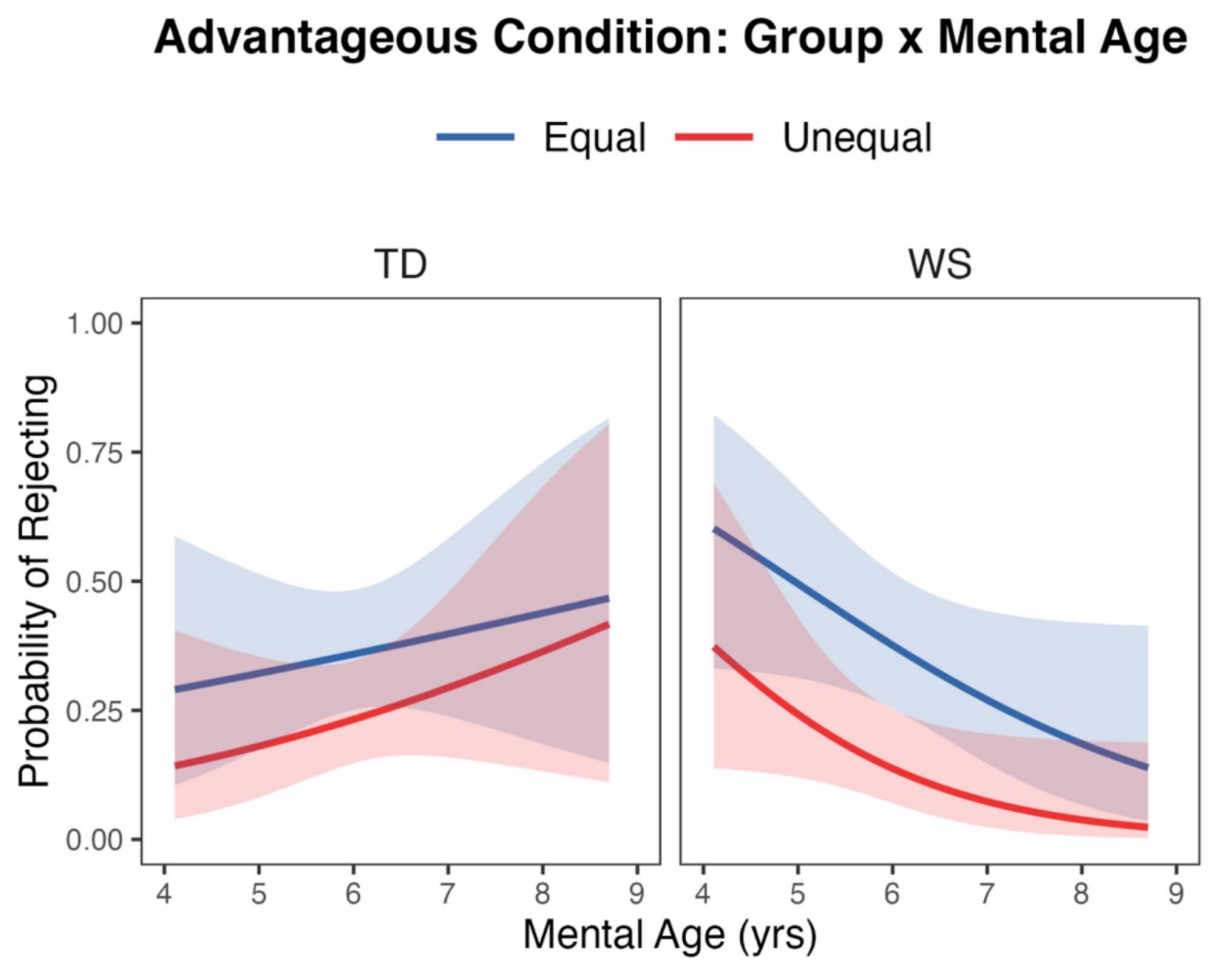
Inequity game, advantageous condition. Interaction of group and mental age, showing equal and unequal trial rejections with 95% confidence intervals

Because older children typically accept both the equal and unequal trials in the Advantageous condition, we conducted exploratory analyses to examine the effects of life experience (indexed by chronological age, CA) and cognitive development (indexed by mental age, MA). The addition of main effects and interactions for CA did not improve model fit compared to the main effects model described above. However, a model including a main effect of MA and an interaction between Group and MA did improve fit: (*LRT* χ^2^ (2) = 6.42, *p* = 0.04). This improvement held when CA was added as a control variable. A simple slopes analysis (Fig. 4) showed that rejections decreased as MA increased for the WS group (-0.54, 95% CI[-0.98, -0.09]) but no change occurred for the TD group (0.24, 95% CI[-0.26, 0.73]).

We also conducted a correlation analysis for MA and CA and rejections by Group, Condition and Distribution (Table 3). There were no significant correlations with CA and only one significant correlation with MA. In the TD group, Disadvantageous condition, rejections of unequal distributions increased with MA (*R* = 0.6, *p* = 0.01).

**Table 3:** Inequity game, correlations with Chronological and Mental age. A. correlations between chronological age and rejections for each group. B. Correlations between mental age and rejections for each group

Group	Advantageous condition		Disadvantageous condition	
	Equal trials	Unequal trials	Equal trials	Unequal trials
**A) Chronological Age**				
WS	*R* = 0.2; *p* = 0.5	*R* = 0.14; *p* = 0.64	*R* = 0.41; *p* = 0.15	*R* = 0.06; *p* = 0.85
TD	*R* = 0.27; *p* = 0.29	*R* = 0.34; *p* = 0.18	*R* = 0.38; *p* = 0.13	*R* = 0.10; *p* = 0.7
**B) Mental Age**				
**WS**	*R* = -0.42; *p* = 0.14	*R* = -0.42; *p* = 0.13	*R* = -0.4; *p* = 0.16	*R* = -0.24; *p* = 0.42
**TD**	*R* = 0.14; *p* = 0.59	*R* = 0.19; *p* = 0.47	*R* = 0.15; *p* = 0.55	***R*** **= 0.6; *p*** **= 0.01**

In a further exploratory analysis, we focused on rejections of the equal trials to assess whether participants were rejecting the 1 reward they could receive in the equal trials after seeing that they could get 4 rewards in the unequal trials. If this were the case, we would expect higher rejections of equal trials if an unequal trial came first compared to when an equal trial came first. Examining these first equal trials showed that this was the case: WS group, 10% rejecting equal when it was the first trial, 25% rejecting equal when an unequal trial came first; TD group, 27% equal first trial, 66% unequal trial first. However, we note that these percentages are skewed because far fewer participants received an unequal trial first (WS, *n* = 4; TD, *n* = 6) compared to having an equal first trial (WS, *n* = 10; TD, *n* = 11). Chi-squared tests were not significant for either group: WS, χ^2^ (1) = 0.53, *p* = 0.46; TD, χ^2^ (1) = 2.49, *p* = 0.11.

## Discussion

The WS and TD groups made similar decisions in both the Disadvantageous and Advantageous conditions of the Inequity Game. For the Disadvantageous condition, both groups followed a pattern found in all groups tested using this task to date: rejecting disadvantageous trials at higher rates compared to equal trials. We had predicted that the WS group would *accept* the disadvantageous trials, or at least reject them at lower rates compared to the TD group, due to a higher motivation to be prosocial towards the recipient. However, instead of using a more simple, “be nice,” approach, the WS group rejected these trials just as the TD group did.

For the Advantageous condition, both the WS and TD groups again made similar decisions, accepting the advantageous offers, but also rejecting a substantial proportion of equal offers. Adding mental age to the regression models revealed that, for the WS group, rejections decreased overall as mental age increased. We consider the implications of this result in the General Discussion.

### General discussion

Social motivation plays a critical role in many behaviors that are important for social life. Recent research with individuals with WS has demonstrated that social motivation without sophisticated social cognition, appears sufficient for some forms of social learning [20, 25–27, 90, 91] and prosociality [31]. The current study adds to this body of research by assessing sharing and fairness in WS and TD individuals. Overall, we find great similarity between the two groups for both sharing and fairness and elevated sharing for the WS group after observing an adult model giving behavior. We consider these results for each experiment separately and then together.

In the sharing task (Experiment 1), the WS and TD groups gave approximately the same amounts in the baseline Dictator Game. This result mirrors those of a study on helping which found no difference between WS and TD samples [31] and suggests that social motivation may be sufficient for sharing at typical levels. However, one potential problem with this proposal is that individuals with ASD also give at the same levels as TD IQ-matched controls [1, 2, 92]. Given that individuals with ASD tend to have lower social motivation than individuals with WS in general [12, 93], the appropriate amount to give in sharing situations may have been learned through observation and/or experience. Indeed, the TD, WS and ASD groups in these studies all kept more resources for themselves, on average. Thus, while it is possible that individuals with WS arrive at the baseline sharing level via social motivation, they might also reach the same decision through learning mechanisms shared with individuals with ASD.

More convincing evidence of the role of social motivation in sharing comes from the modeling conditions. The WS participants gave more than their baseline after exposure to both the generous and selfish models, and the WS group was more likely than the TD group to give more than half, across all conditions. These results support the role of social motivation for giving, but in a limited way. In this experimental design, participants do not give directly to the recipient but rather place the donated rewards into a box. In addition, in the modeling conditions, the experimenter turns away while the participant gives rewards. Therefore, there are no social rewards from giving directly and seeing either the recipient’s or the modeler’s positive response. That said, participants likely found the social interaction in the modeling conditions more rewarding than interaction in the baseline condition. Observing the adult models give something to the recipient (either 1 or 5, depending on condition) may have provided a socially rewarding experience that increased the observer’s generosity. Observing the models give may also have signaled to the participants that giving was socially rewarding and that this social reward outweighed the value of the candies. These possibilities are in line with a recent study showing that, for WS participants, social stimuli have a high intrinsic reward value [91].

Intriguingly, individuals with WS were not motivated to imitate the adult models exactly. When the adult model was selfish and gave only 1, WS participants did not lower their baseline giving to match that donation level. Rather, they gave more to the recipient compared to their baseline. For the Selfish condition, the WS group may have viewed the donation of 1 as a “nice” act and then responded by giving more than they typically would. If true, then the WS participants would not have been motivated to imitate per se but rather motivated to engage in more generous behavior. This possibility also suggests that the TD group lacks that specifically generous motivation or they may engage in more sophisticated social cognition when deciding not to follow the generous model. For example, the TD children may use a norm of equal giving strategically and reason that giving half is enough [51].

In the fairness task (Experiment 2), WS and TD participants received both disadvantageous and advantageous conditions and made similar decisions. For the disadvantageous condition, both groups rejected an offer of 1 for the participant and 4 for the recipient more than they rejected equal (1 for each) offers. This pattern of rejections requires a comparison of the quantity of treats given to oneself and the recipient. For both equal and unequal trials in this condition, the participant receives 1 treat, but in the unequal trials the recipient receives 4 compared to 1 in the equal trials. Rejecting the unequal trials more than the equal trials thus demonstrates that the participant is engaging in a social and quantitative comparison. This result also shows that participants in both the WS and TD groups had the self-control to resist the temptation to receive the one treat they would receive from accepting the offer. In addition to these cognitive and behavioral skills, rejecting a disadvantageous offer also requires social motivation, albeit a negative one. Multiple studies have demonstrated that TD populations reject a disadvantage in order to prevent a peer from getting more [94, 95]. We had predicted that individuals with WS would accept the disadvantageous offers, thus delivering 4 treats to the recipient and revealing a prosocial motivation. The fact that the WS group rejected these offers suggests they share the same self-focused social motivation for fairness as the TD group [57].

A more positive form of fairness also requires sacrificing one’s rewards but with a goal of preventing the recipient from getting less. Rejecting an advantage is other-focused and requires more sophisticated social cognition including recognizing a general norm of fairness as equity, an understanding that receiving less will make the recipient sad, and the self control required to reject a relatively large reward [57]. Neither the TD group, 6-year-olds, nor the WS group rejected the advantage more than the equal trials and in fact both groups were more likely to reject the equal offers. This pattern of rejections has been found for TD children around 4 to 5 years of age in some studies, but for the most part children between 4 and 7 years of age accept both advantageous and equal trials [61]. By about 8 to 10 years of age, children in several countries reject the advantage and accept the equal trials [62].

It is possible that participants deviated from typical behavior in the advantageous condition either because they lacked relevant life experience (proxied by chronological age) or more sophisticated cognitive abilities (measured by mental age). In fact, despite having a much wider age range in the WS group, chronological age (CA) did not predict rejections in this condition. In contrast, mental age (MA), a reflection of more general cognitive development, did predict rejections for the WS group: higher MA predicted fewer rejections of both equal and unequal trials. There was no change by MA for the TD group. This suggests that the acquisition of complex cognitive abilities, such as inhibitory control and other executive functions, may be required for individuals with WS to disengage from a focus on their own payoffs and accept more of the equal trials.

A second consideration focuses on the rejections of the equal trials in the advantageous condition. Participants may have simply focused on their own rewards, accepting the offers of 4 for themselves and rejecting when the offer is 1. Rejections in this case may have been due to frustration at not getting the larger amount they had received before. Some evidence for this explanation comes from comparing rejections of the first equal trials participants received based on whether this was the first trial they received or a subsequent trial preceded by an unequal trial. For both the WS and the TD group, the first equal trial was more likely to be rejected when preceded by an unequal trial. Although this contrast was not significant for either group, the effect was stronger for the TD group suggesting a strategy of signaling a desire for the larger reward in the unequal trials. Future experiments can limit this issue by making the quantities for the equal offers match the advantaged amount thus removing any change in the participant’s rewards.

Considering both experiments together offers insight both into the role of social motivation in sharing and fairness and into the abilities of individuals with WS. For baseline sharing and disadvantageous inequity aversion, the WS and TD groups made similar decisions. For these “typical” behaviors, social motivations without sophisticated social cognition may be sufficient: altruistic, but not generous, motivations for sharing and a motivation to prevent one from being at a disadvantage. Both behaviors suggest a role for equality as a limit on resource distribution. However, a generous motivation can override this limit for the WS group in the case of sharing when an adult model is observed giving anything to a recipient. Although giving more than half has been found in some societies in similar experiments, it remains rare for TD children in Western societies [51].

For advantageous inequity aversion, neither the WS nor TD group were willing to reject an advantage more than the equal trials. Our limited evidence suggests that more advanced cognitive development may be required for WS participants to show the typical pattern in this condition, and that TD participants may have focused only on their own rewards. Nevertheless, the fact that the results of the advantageous condition differed markedly from those in the disadvantageous condition fits with the theoretical view that these two forms of fairness are supported by different cognitive and motivational processes [57].

The current study also reveals a more complex behavioral profile for individuals with WS. Although the WS phenotype highlights social motivation, those motivations are not simply prosocial. The WS group gave similar amounts in the baseline sharing task and gave more generously than the TD group after viewing adult models, behaviors that are prosocial. However, when faced with a disadvantageous offer, the WS group rejected the offer and denied rewards to the recipient. This result aligns with TD behaviors for disadvantageous inequity aversion, an early emerging and self-focused form of fairness [57]. While this is not a prosocial behavior, it is typical behavior and a normal form of social interaction that may help individuals with WS navigate situations in which they might be treated unfairly.

### Limitations

Although to the best of our knowledge the present study is the first experimental investigation of sharing and fairness-related behaviors in WS, there are limitations which caution against generalizing the results. First, as often happens in research with rare populations such as WS, the sample size was small and precluded investigating gender differences. Second, our study adopted a MA-matched design that, although it is one of the most commonly used matching approaches, increases the chronological age discrepancy between groups. We are aware that this discrepancy can be a limit in interpreting the results when exploring a domain in which amount of social experience could play a role, such as in sharing and fairness behaviors. Future studies could use a more comprehensive experimental design including another group matched on MA and CA with the WS group.

Another limitation applies to the experiments themselves. Although we argue that the Dictator Game and the Inequity Game assess giving and fairness behaviors, respectively, neither experiment used a real recipient. In the DG, the recipient was a hypothetical child or person that the experimenter would see later. In the IG, the recipient was a puppet being manipulated by another experimenter. While these artificial recipients may have decreased the external validity of the tasks, they also likely underestimated the effects found for both experiments. The emotional reactions of a live recipient would likely have increased both giving and fairness behaviors, and these increases would likely be greater in the WS sample. This limitation could be addressed in future studies by either adding happy or sad emotions and vocalizations to recipient puppets or by comparing a puppet to a live recipient.

## Conclusions

In summary, individuals with WS appear capable of both preventing disadvantageous outcomes and of learning to be more generous. These abilities suggest greater competence in navigating social environments than has previously been shown. In addition, the current study also provides evidence that social motivation without sophisticated social cognition may be sufficient for sharing and, in particular, generous sharing, as well as the self-focused form of fairness.

### Electronic supplementary material

Below is the link to the electronic supplementary material.

Supplementary Material 1

Supplementary Material 2

Supplementary Material 3

Supplementary Material 4

Supplementary Material 5

## Data Availability

All data generated or analysed during this study are included in this published article as supplementary information files.

## Abbreviations

TD: Typically developing
WS: Williams syndrome
DG: Dictator Game
IG: Inequity Game
ASD: Autism spectrum disorder
DS: Down syndrome
MA: Mental age
IQ: Intelligence quotient
CA: Chronological age
LRT: Likelihood ratio tests
